# Characteristics and risk factors for mortality in patients with acute coronary syndrome concomitant sepsis: a retrospective multicenter cohort study

**DOI:** 10.3389/fcvm.2025.1703505

**Published:** 2025-11-18

**Authors:** Yinuo Zhu, Lei Wang, Yan Liu, Guoying Zheng, Ming Wu, Zhifeng Liu, Jinxia Zhang

**Affiliations:** 1 The First School of Clinical Medicine, Southern Medical University, Guangzhou, China; 2 Department of Cardiology, General Hospital of Southern Theatre Command of PLA, Guangzhou, China; 3 High Dependency Unit, Department of Critical Care Medicine, Zhanjiang Central People’s Hospital, Zhanjiang, China; 4 Hospital-Acquired Infection Control Department, Shenzhen Second People's Hospital, Shenzhen, China; 5 Department of Critical Care Medicine, Huadu District People’s Hospital, Guangzhou, China; 6 Department of Medicine Critical Care Medicine, General Hospital of Southern Theatre Command of PLA, Guangzhou, China

**Keywords:** acute coronary syndrome, sepsis, risk factors, nomogram, acute myocardial infarction

## Abstract

**Objective:**

The purpose of this research was to examine the risk factors associated with in-hospital mortality in patients with acute coronary syndrome (ACS) concomitant sepsis, and to develop and verify a nomogram model for predicting mortality risk.

**Methods:**

This multicenter retrospective analysis examined clinical data from patients with ACS concomitant sepsis who were hospitalized in the intensive care units of tertiary hospitals in Southern China between January 2013 and December 2023. In-hospital mortality functioned as the principal outcome. Univariate and multivariate logistic regression analysis, together with LASSO regression, were used to ascertain independent risk factors for the outcome. The evaluation of model performance was conducted by receiver operating characteristic (ROC) curves, area under the curve (AUC), and calibration plots.

**Results:**

This study comprised a total of 200 patients. During hospitalization, 122 people (61.0%) succumbed. Multivariate logistic regression analysis indicated that the diagnosis of ST-segment elevation myocardial infarction (STEMI) at admission (OR = 2.081, 95% CI: 1.120–3.866, *P* = 0.0206), an elevated initial neutrophil count (OR = 1.05, 95% CI: 1.000–1.102, *P* = 0.0495), and a history of coronary artery disease (OR = 2.953, 95% CI: 1.173–7.436, *P* = 0.0215) were independent risk factors for in-hospital mortality. The nomogram model that includes these parameters exhibited an AUC of 0.641 (95% CI: 0.564–0.718), with a sensitivity of 0.656 and a specificity of 0.603. Calibration curves demonstrated strong concordance between expected and observed results (Hosmer-Lemeshow test *P* > 0.05).

**Conclusion:**

Patients with ACS concomitant sepsis experience heightened in-hospital mortality, which is substantially correlated with a diagnosis of STEMI at admission, increased initial neutrophil count, and pre-existing coronary artery disease. While the discriminative capacity (AUC = 0.641) of this three-factor nomogram necessitates additional enhancement, its commendable calibration provides a first instrument for early risk categorization, illustrating practical applicability for swift evaluation. Extensive investigations are necessary to improve model efficacy.

## 1 Introduction

Cardiovascular diseases (CVD) exhibit an escalating prevalence around the world, constituting the predominant etiology of mortality in both urban and rural demographics. Acute coronary syndrome (ACS), representing a paramount life-threatening manifestation of CVD, is responsible for 18%–23% of CVD-associated fatalities (1), demonstrating in-hospital mortality rates ranging from 1.8% to 15.7% (2–4). Sepsis, characterized by a maladaptive host response to infection, exhibits in-hospital mortality rates approaching 33% (5). Of particular significance is the intricate bidirectional pathophysiological relationship between sepsis and ACS, which may establish a self-perpetuating pathogenic cycle upon co-occurrence. Epidemiological evidence identifies sepsis and septic shock as the predominant non-cardiac causes of mortality in acute myocardial infarction (AMI) patients receiving percutaneous coronary intervention (PCI) (6). Approximately 7% of acute myocardial infarction patients develop secondary sepsis, resulting in a one-year mortality rate up to 70% (far higher than the combined mortality rate of two independent diseases), and their long-term survival rates are much inferior to those without sepsis (2).Patients with ACS exhibit varying degrees of myocardial ischemia. The ensuing release of damage-associated molecular patterns (DAMPs) during ischemic events potentiates systemic inflammatory cascades through TLR4/NF-κB pathway activation (7, 8). Consequent hemodynamic derangements precipitate intestinal barrier dysfunction, thereby elevating susceptibility to microbial translocation with subsequent hematogenous dissemination of enteric bacteria and their metabolic byproducts. Following translocation, pathogen-derived components (e.g., lipopolysaccharide) markedly augment both localized and systemic inflammatory responses through pattern recognition receptor (PRR) activation, culminating in endogenously seeded infections, endotoxemia, and progressive evolution to multiple organ dysfunction syndrome (MODS) (9–11).

The pathophysiological progression of sepsis is characterized by three interconnected processes that significantly impact cardiovascular homeostasis: exaggerated proinflammatory cytokine production, endothelial dysfunction, and dysregulated coagulation cascades (12–14). These pathobiological mechanisms collectively contribute to atherosclerotic plaque destabilization and potentiate acute thrombotic events. The systemic inflammatory milieu associated with sepsis exacerbates coronary plaque vulnerability through multiple pathways, including but not limited to matrix metalloproteinase (MMP)-mediated extracellular matrix degradation, enhanced platelet activation, and coagulation pathway disturbances (15, 16). Furthermore, circulating endotoxins exert direct cardiotoxic effects by disrupting mitochondrial bioenergetics and calcium handling mechanisms, culminating in septic cardiomyopathy (SICM) (17–19). Concurrent hemodynamic instability, manifesting as tachycardia and hypotension, further compromises myocardial oxygen delivery-demand equilibrium (20, 21).

Contemporary registry data indicate that acute coronary syndrome (ACS) patients complicated by severe infections experience mortality rates approaching 23%, representing a fourfold increase compared to their non-infected counterparts (22). The APEX-AMI trial provided further validation of this association, demonstrating that 29% of ST-segment elevation myocardial infarction (STEMI) patients developed nosocomial infections during index hospitalization, with attendant mortality risks nearly an order of magnitude greater than uncomplicated STEMI cases (23).

Notwithstanding established associations between infectious processes and cardiovascular events, several knowledge gaps persist regarding sepsis-mediated impacts on ACS outcomes. Consequently, an integrated tool incorporating both cardiovascular and infectious parameters is urgently needed to address this clinical dilemma. Current prognostic instruments exhibit notable limitations in clinical applicability. Conventional risk stratification tools maintain disease-specific orientations, failing to account for the bidirectional pathophysiological interplay between sepsis and ACS. The Sequential Organ Failure Assessment (SOFA) score, while valuable for quantifying organ dysfunction, demonstrates limited predictive capacity for ACS-specific endpoints such as plaque rupture or recurrent infarction. Conversely, established cardiovascular risk scores (GRACE, TIMI) inadequately incorporate infection-related prognostic variables. Existing literature also reveals significant population heterogeneity, with non-ST-segment elevation myocardial infarction (NSTEMI) and unstable angina (UA) cohorts being particularly underrepresented in sepsis-ACS investigations, despite their heightened inflammatory profiles and potential infection susceptibility. While emerging biomarkers (e.g., S100A8/A9, resistin) show diagnostic promise, their clinical implementation remains constrained by analytical requirements. Similarly, novel noninvasive monitoring modalities, despite demonstrating utility in septic shock prognostication, currently lack validated parameters for assessing coronary-specific pathology in ACS patients.

To bridge these translational gaps, we conducted a multicenter retrospective cohort study examining patients with ACS concomitant sepsis. Our investigation systematically characterized this high-risk population's clinical phenotype, elucidated independent risk factors, and developed a comprehensive nomogram-based predictive algorithm. This integrated risk stratification tool facilitates early identification of high-mortality-risk patients, enabling timely therapeutic intervention and potentially improving clinical outcomes.

## 2 Materials and methods

### 2.1 Study population

This research is structured as a multicenter, retrospective cohort analysis. Patients who satisfied the specified inclusion and exclusion criteria and were admitted to the intensive care units (ICUs) of tertiary hospitals in various cities in southern China from January 2013 to December 2023 were enrolled.

The inclusion criteria were as follows: (1) Adult patients (≥18 years old); (2) a definitive diagnosis of ACS established according to the ACS management guidelines published by the European Society of Cardiology (ESC) in October 2023 (24); (3) the diagnostic criteria for sepsis were based on the latest definition jointly issued by the Society of Critical Care Medicine (SCCM) and the European Society of Intensive Care Medicine (ESICM) in 2016, which defines sepsis as life-threatening organ dysfunction caused by a dysregulated host response to infection, which can be associated with a Sepsis-related Organ Failure Assessment (SOFA) score ≥2 points consequent to the infection (25).

The exclusion criteria were as follows: (1) Pregnant or breastfeeding patients; (2) Patients on long-term immunosuppressive therapy or with severe immunodeficiency; (3) Patients with advanced cancer; (4) Patients with irreversible conditions or in a terminal phase; (5) Patients with pre-existing infections prior to acute myocardial infarction onset; (6) Patients who declined treatment during hospitalization; (7) Patients who had received bone marrow or organ transplants.

### 2.2 Grouping criteria

Patients were classified into the death group and the survival group according to their in-hospital outcomes.

### 2.3 Data collection

Data were mostly gathered by examining the electronic medical records system and nursing documentation systems from each participating center. All patients receiving invasive diagnostic or therapeutic procedures, including coronary angiography and intervention, provided signed informed permission.

The primary data gathered encompassed age, gender, medical history, laboratory test results, electrocardiographic findings, and medication history. Laboratory data encompassed initial clinical parameters recorded during 24 h of admission and the first test results obtained within 24 h of a confirmed sepsis diagnosis.

### 2.4 Statistical analysis

Data were examined and processed with R 4.3.3 software. Statistical significance was established at *P* < 0.05 (two-tailed test). Continuous variables with normally distributed data were expressed as mean ± standard deviation, whereas non-normally distributed data were conveyed as median with interquartile range. Comparisons between groups were performed with Student's *t*-test, Mann–Whitney *U*-test, or Kruskal–Wallis *H*-test. For categorical variables, data were reported as proportions and percentages, with group comparisons conducted using Pearson's chi-square test or Fisher's exact test. A systematic study was performed using univariate logistic regression, LASSO regression, and multivariate logistic regression to ascertain independent risk factors.

## 3 Results

### 3.1 Comparison of baseline characteristics between the death group and the survival groups

**Figure 1:**
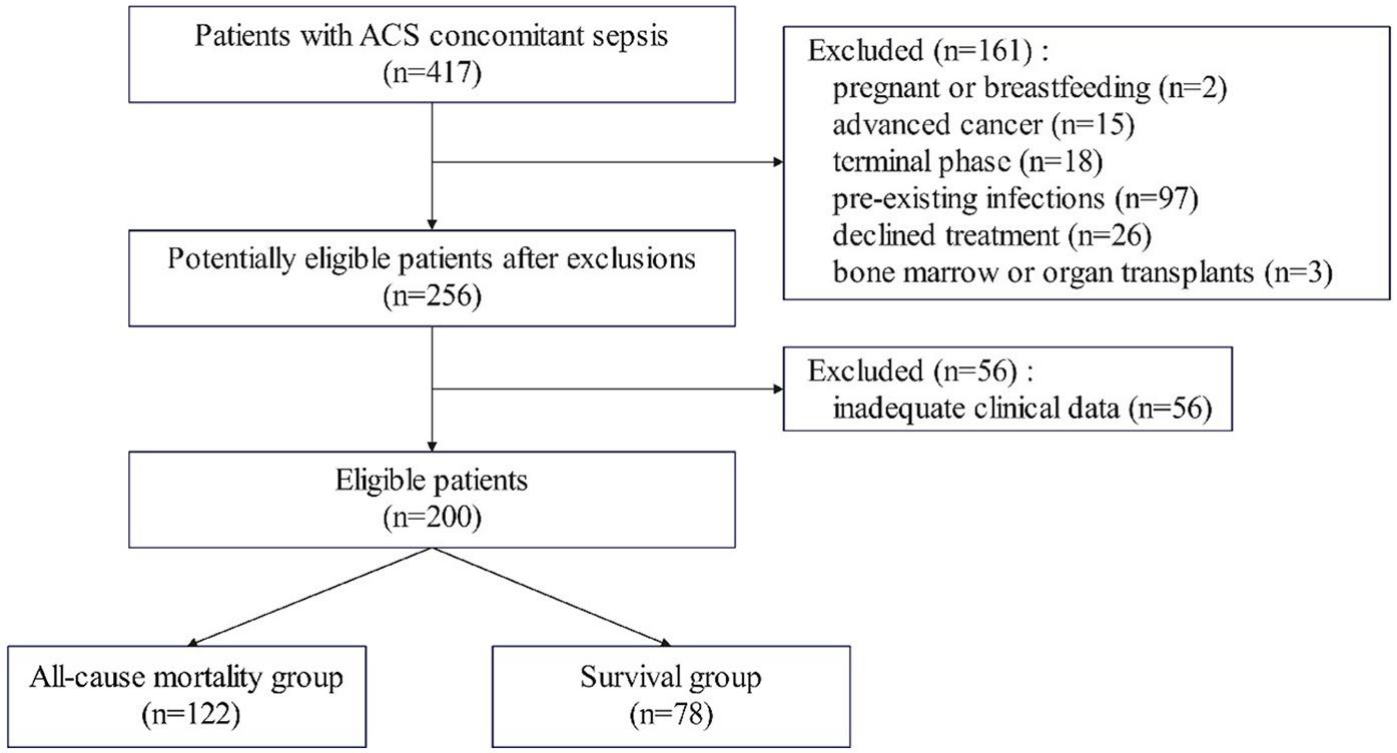
Flowchart of patient screening and enrollment.

A total of 256 patients with ACS concomitant sepsis were initially screened, of which 56 were eliminated due to inadequate clinical data, resulting in the enrollment of 200 patients. Patients were categorized into death (*n* = 122) and survival (*n* = 78) groups based on in-hospital outcomes (Figure 1). No statistically significant differences were detected between the groups for age or gender. The mortality group exhibited a markedly elevated prevalence of pre-existing coronary artery disease (*P* = 0.036) (Table 1).

**Table 1:** Comparison of the baseline characteristics on initial admission between the survival and death groups.

Variables	Total (*n* = 200)	Died (*n* = 78)	Survived (*n* = 122)	*P* Value
DIG (%)				0.117
STEMI	113 (56.5)	38 (48.7)	75 (61.5)	
NSTEMI	22 (11.0)	8 (10.3)	14 (11.5)	
UA	65 (32.5)	32 (41.0)	33 (27.0)	
Age (years)	68.1 (13.6)	67.4 (13.6)	68.6 (13.7)	0.542
Gender (%)				0.167
Male	158 (79.0)	66 (84.6)	92 (75.4)	
Female	42 (21.0)	12 (15.4)	30 (24.6)	
Past history(%)	16 (8.0)	6 (7.7)	10 (8.2)	
Smoking	97 (48.5)	37 (47.4)	60 (49.2)	0.2
HBP	68 (34.0)	23 (29.5)	45 (36.9)	0.924
DM	13 (6.5)	2 (2.6)	11 (9.0)	0.355
Gastric disease	5 (2.5)	0 (0.0)	5 (4.1)	0.131
CHF	22 (11.0)	7 (9.0)	15 (12.3)	0.178
CKD	6 (3.0)	2 (2.6)	4 (3.3)	0.617
Stroke	16 (8.0)	7 (9.0)	9 (7.4)	0.312
COPD	113 (56.5)	38 (48.7)	75 (61.5)	0.889
CAD	33 (16.5)	7 (9.0)	26 (21.3)	**0**.**036**
Arrhythmia	3 (1.5)	0 (0.0)	3 (2.5)	0.424
VHD	5 (2.5)	1 (1.3)	4 (3.3)	0.676
AMI	17 (8.5)	5 (6.4)	12 (9.8)	0.557
PCI	25 (12.5)	7 (9.0)	18 (14.8)	0.324
Stent placement	16 (8.0)	5 (6.4)	11 (9.0)	0.693
SBP (mmHg)	105.0 (35.5)	104.6 (39.2)	105.2 (33.1)	0.91
DBP (mmHg)	61.8 (22.3)	61.5 (24.3)	62.0 (21.0)	0.88
HR (bpm)	81.7 (30.8)	76.3 (31.3)	85.1 (30.1)	**0**.**049**
R (bpm)	21.5 (3.8)	21.6 (3.8)	21.5 (3.7)	0.848
T (°C)	36.3 (0.7)	36.2 (0.7)	36.4 (0.8)	0.055
RBC (10^12^/L)	4.4 (8.6)	3.8 (1.1)	4.8 (11.0)	0.409
Hb (g/dL)	113.8 (32.7)	113.8 (34.8)	113.8 (31.5)	0.998
WBC (10^9^/L)	12.2 [7.2, 16.6]	10.3 [6.3, 15.1]	12.7 [7.6, 17.2]	0.081
NEUT (10^9^/L)	9.6 [4.6, 14.2]	8.4 [4.0, 12.7]	10.6 [5.6, 14.6]	**0**.**03**
LYMPH (%)	1.1 [0.4, 1.8]	1.2 [0.3, 2.0]	1.1 [0.5, 1.7]	0.529
MONO (%)	0.5 [0.2, 0.9]	0.5 [0.1, 0.7]	0.6 [0.3, 0.9]	0.143
PLT (10^9^/L)	194.0 [123.5, 252.2]	194.5 [118.8, 225.2]	193.0 [124.0, 268.5]	0.358
Alb (g/dL)	35.0 [19.9, 39.6]	35.8 [19.9, 40.6]	34.7 [20.0, 39.1]	0.658
Cr (mg/dL)	97.5 [64.5, 148.2]	88.5 [55.5, 132.5]	105.3 [70.6, 158.0]	0.08
BG (mg/dL)	9.5 [5.9, 15.1]	8.1 [5.5, 12.7]	10.0 [6.2, 16.5]	0.095
TB (mg/dL)	9.6 [4.8, 14.2]	8.7 [1.7, 14.0]	10.5 [6.5, 14.1]	0.223
UA (mg/dL)	380.5 [194.5, 493.2]	350.0 [114.5, 452.0]	405.5 [206.0, 518.5]	0.074
ALT (U/L)	34.0 [1.0, 76.1]	38.0 [1.0, 92.1]	32.1 [1.0, 65.5]	0.402
APTT (s)	36.4 [27.6, 43.5]	34.1 [26.7, 41.3]	37.2 [28.3, 49.8]	0.151
INR	1.0 [0.9, 1.2]	1.0 [0.9, 1.2]	1.1 [0.9, 1.3]	0.166
Fib (mg/dL)	3.3 [1.9, 4.5]	2.8 [1.7, 4.1]	3.5 [2.2, 4.6]	0.161
K (mmol/L)	3.8 (0.9)	3.7 (0.9)	3.9 (1.0)	0.28
Na (mmol/L)	134.4 (9.8)	133.7 (9.9)	134.9 (9.7)	0.392
Cl (mmol/L)	97.4 (10.1)	97.5 (10.6)	97.4 (9.8)	0.963

HBP, high blood pressure; DM, diabetes mellitus; CHF, congestive heart failure; CKD, chronic kidney disease; COPD, chronic obstructive pulmonary disease; VHD, valvular heart disease; SBP, systolic blood pressure; DBP, diastolic blood pressure; HR, heart rate; R, respiratory rate; T, temperature; RBC, red blood cell count; Hb, hemoglobin; WBC, white blood cell count; NEUT, neutrophil count; LYMPH, lymphocyte percentage; MONO, monocyte percentage; PLT, platelet count; Alb, albumin; Cr, creatinine; BG, blood glucose; TB, total bilirubin; UA, uric acid; ALT, alanine aminotransferase; APTT, activated partial thromboplastin time; INR, international normalized ratio; Fib, fibrinogen; K, potassium; Na, sodium; Cl, chloride. Bold values in the table indicate statistical significance (*P* < 0.05).

Preliminary laboratory assessments at entry indicated that the death group demonstrated markedly elevated heart rates (*P* = 0.049) and neutrophil counts (*P* = 0.03) in comparison to the survival group (Table 1).

In the context of diagnostic tests for sepsis confirmation, the death group exhibited significantly increased levels of activated partial thromboplastin time (*P* = 0.04), blood glucose (*P* = 0.009), creatine kinase (*P* = 0.016), anion gap (*P* = 0.008), serum sodium (*P* = 0.028), and peak procalcitonin (*P* < 0.001) (Table 2).

**Table 2:** Comparison of laboratory findings at diagnosis of sepsis and in-hospital therapies/complications between the survival and death groups.

Variables	Total (*n* = 200)	Died (*n* = 78)	Survived (*n* = 122)	*P* Value
SBP (mmHg)	110.8 (26.2)	114.7 (27.2)	108.3 (25.3)	0.09
DBP (mmHg)	63.7 (14.8)	65.6 (14.8)	62.5 (14.6)	0.148
HR (bpm)	98.2 (23.1)	96.8 (22.6)	99.2 (23.5)	0.475
T (℃)	37.1 (2.2)	37.3 (1.8)	36.9 (2.5)	0.297
RBC (10^12^/L)	3.9 (0.9)	3.9 (0.8)	3.8 (1.0)	0.858
Hb (g/dL))	114.3 (25.9)	116.3 (26.4)	113.0 (25.6)	0.382
WBC (10^9^/L)	15.8 [11.7, 19.8]	15.3 [10.0, 19.3]	16.0 [12.5, 20.5]	0.179
NEUT (10^9^/L)	13.5 [9.8, 17.4]	13.1 [8.2, 16.9]	13.9 [10.3, 18.1]	0.177
LYMPH (%)	1.0 [0.6, 1.5]	1.1 [0.6, 1.6]	1.0 [0.6, 1.4]	0.707
MONO (%)	0.8 [0.6, 1.2]	0.8 [0.5, 1.2]	0.9 [0.6, 1.2]	0.774
PLT (10^9^/L)	160.5 [120.8,244.8]	165.0 [128.8,247.0]	160.0 [112.0,237.2]	0.367
APTT (seconds)	45.8 [39.0, 61.7]	43.6 [37.9, 53.9]	47.3 [39.5, 65.3]	**0**.**04**
D-Dimer (μg/mL)	3.2 [1.4, 6.6]	2.5 [1.1, 5.8]	3.6 [1.6, 7.0]	0.105
INR	1.3 [1.1, 1.7]	1.3 [1.1, 1.6]	1.4 [1.2, 1.8]	0.11
Fig (mg/dL)	4.9 [3.3, 6.3]	4.2 [3.2, 6.3]	5.0 [3.4, 6.5]	0.278
PCT (ng/mL)	3.4 [0.7, 13.3]	2.2 [0.5, 9.0]	4.2 [0.8, 17.2]	0.068
max PCT (ng/mL)	12.4 [3.6, 30.9]	6.2 [1.7, 20.1]	17.7 [6.0, 40.4]	**0**.**000047**
CK-MB (ng/mL)	32.0 [10.0, 101.0]	27.2 [10.1, 112.7]	39.7 [10.2, 92.7]	0.693
Max CK-MB (ng/mL)	61.0 [16.3,187.8]	56.9 [16.8,159.9]	66.9 [16.1,194.7]	0.846
Alb (g/dL)	32.9 (5.7)	33.9 (5.3)	32.3 (5.8)	0.053
Cr (mg/dL)	177.5 [121.0,272.2]	154.5 [114.0,264.7]	180.2 [130.2,272.8]	0.217
BG (mmol/L)	9.6 [7.6, 14.1]	8.9 [7.1, 10.9]	10.4 [8.2, 15.0]	**0**.**009**
TC (mg/dL)	4.0 [3.3, 5.0]	4.0 [3.3, 5.2]	4.2 [3.2, 5.0]	0.771
TG (mmol/L)	1.2 [0.8, 1.7]	1.1 [0.8, 1.6]	1.3 [0.8, 1.8]	0.27
TB (mg/dL)	14.9 [9.4, 25.0]	13.8 [8.8, 23.3]	15.9 [10.1, 26.1]	0.218
UA (mg/dL)	437.0 [297.9,600.0]	414.5 [297.0,540.5]	453.5 [299.2, 617.8]	0.408
ALT (U/L)	104.0 [38.8, 538.9]	90.5 [39.2, 229.5]	126.7 [38.2, 937.3]	0.244
AST (U/L)	218.0 [77.0,802.8]	178.0 [66.8,567.8]	258.0 [84.0, 986.2]	0.106
CK (U/L)	1,015.5 [284.0, 3464.2]	638.5 [205.0, 2728.5]	1,519.5 [364.2, 4206.8]	**0**.**016**
Blood pH	7.4 (0.1)	7.4 (0.1)	7.3 (0.2)	0.155
PaO_2_ (mmHg)	99.2 [81.0, 131.2]	100.0 [82.1,130.8]	98.8 [80.2, 132.2]	0.992
PaCO_2_ (mmHg)	36.0 [30.8, 44.3]	36.8 [30.8, 44.6]	35.3 [30.8, 42.7]	0.738
Oxygen Saturation (%)	95.5 (8.6)	95.3 (12.0)	95.6 (5.7)	0.798
Bicarbonate (mEq/L)	20.7 [16.2, 25.4]	21.6 [16.5, 25.7]	20.5 [16.2, 24.8]	0.57
Base excess (mEq/L)	6.0 [2.3, 9.6]	5.1 [2.0, 9.2]	6.1 [2.8, 9.8]	0.337
Anion Gap (mEq/L)	15.3 [10.9, 20.0]	13.4 [10.0, 16.8]	16.5 [12.0, 21.3]	**0**.**008**
Potassium (mmol/L)	4.2 (0.7)	4.1 (0.7)	4.3 (0.8)	0.103
Sodium (mmol/L)	141.0 [138.0,146.0]	139.1 [137.4,143.9]	142.0 [138.0,146.5]	**0**.**028**
Chloride (mmol/L)	103.8 (11.2)	102.9 (7.5)	104.3 (13.1)	0.387
Phosphate (mg/dL)	1.2 [0.8, 1.6]	1.1 [0.7, 1.4]	1.3 [0.9, 1.8]	**0**.**013**
Lactate (mmol/L)	3.1 [2.1, 6.2]	3.0 [2.1, 5.1]	3.2 [1.9, 6.9]	0.525
Therapies				
PCI this time (%)	141 (70.5)	59 (75.6)	82 (67.2)	0.265
Thrombosis (%)	9 (4.5)	2 (2.6)	7 (5.7)	0.48
Pacemaker (%)	17 (8.5)	7 (9.0)	10 (8.2)	1
IABP (%)	57 (28.5)	17 (21.8)	40 (32.8)	0.129
Bronchoscopy (%)	54 (27.0)	21 (26.9)	33 (27.0)	1
Dialysis (%)	79 (39.5)	22 (28.2)	57 (46.7)	**0**.**014**
Transfusion (%)	86 (43.0)	19 (24.4)	67 (54.9)	**0**.**000021**
Tracheotomy (%)	22 (11.0)	7 (9.0)	15 (12.3)	0.617
Res-T(h)	126.7[39.3,281.0]	89.3[13.0,239.7]	158.4[56.9,295.6]	**0**.**035**
Beta-blockers (%)	102 (51.0)	47 (60.3)	55 (45.1)	0.051
Statins (%)	183 (91.5)	73 (93.6)	110 (90.2)	0.557
Antiplatelet Medications (%)	193 (96.5)	77 (98.7)	116 (95.1)	0.332
OAC (%)	7 (3.5)	1 (1.3)	6 (4.9)	0.332
Intravenous Anticoagulants (%)	153 (76.5)	59 (75.6)	94 (77.0)	0.954
PPI (%)	183 (91.5)	72 (92.3)	111 (91.0)	0.946
Insulin (%)	71 (35.7)	29 (37.2)	42 (34.7)	0.839
OHA (%)	36 (18.0)	14 (17.9)	22 (18.0)	1
Vasopressor Agents (%)	168 (84.0)	53 (67.9)	115 (94.3)	**7.3835** **×** **10^−7^**
NE (%)	78 (39.0)	20 (25.6)	58 (47.5)	**0**.**003**
NE Dose	0.7 [0.4, 1.3]	0.7 [0.5, 1.3]	0.7 [0.4, 1.2]	0.478
Days of Antibiotic Use	10.0 [5.0, 16.0]	13.0 [9.0, 18.8]	8.0 [4.0, 14.0]	**0**.**000396**
Complications				
HospitalizationTime (days)	13.0 [7.0, 21.2]	19.5 [13.0, 26.0]	10.0 [5.2, 18.0]	**6.7804** **×** **10^−8^**
ICU Time (days)	1.0 [0.0, 3.0]	1.0 [0.0, 2.0]	1.0 [0.0, 3.8]	0.235
Cardiac Arrest (%)	50 (25.0)	15 (19.2)	35 (28.7)	0.181
Lung Complications (%)	93 (46.5)	41 (52.6)	52 (42.6)	0.219
Liver Complications (%)	91 (45.5)	25 (32.1)	66 (54.1)	**0**.**004**
Kidney Complications (%)	113 (56.5)	37 (47.4)	76 (62.3)	0.055
Bleeding Complications (%)	50 (25.0)	14 (17.9)	36 (29.5)	0.094
Aneurysm (%)	3 (4.3)	7 (6.5)	0.083	0.773
Shock (%)	116 (58.0)	28 (35.9)	88 (72.1)	**4.1073** **×** **10^−7^**

SBP, systolic blood pressure; DBP, diastolic blood pressure; HR, heart rate; T, temperature; RBC, red blood cell count; Hb, hemoglobin; WBC, white blood cell count; NEUT, neutrophil percentage; LYMPH, lymphocyte percentage; MONO, monocyte percentage; PLT, platelet count; APTT, activated partial thromboplastin time; INR, international normalized ratio; Fib, fibrinogen; PCT, procalcitonin; max PCT, maximum procalcitonin; CK-MB, creatine kinase-MB; Alb, albumin; Cr, creatinine; BG, blood glucose; TC, total cholesterol; TG, triglycerides; TB, total bilirubin; UA, uric acid; ALT, alanine aminotransferase; AST, aspartate aminotransferase; CK, creatine kinase; Blood pH, blood pH; PaO2, partial pressure of oxygen; PaCO2, partial pressure of carbon dioxide; Res-T, duration of mechanical ventilation; OAC, oral anticoagulant; PPI, proton pump inhibitor; OHA, oral hypoglycemic agent; NE, norepinephrine.

Bold values in the table indicate statistical significance (*P* < 0.05).

### 3.2 Comparison of clinical characteristics between the death group and the survival groups

Analysis of pharmacological interventions and invasive procedures indicated that the death group underwent significantly greater hemofiltration (*P* = 0.014), blood transfusions (*P* < 0.001), vasopressor administration (*P* < 0.001), and norepinephrine treatment (*P* = 0.003), alongside extended mechanical ventilation durations (*P* = 0.035) in comparison to survivors (Table 2). The death group exhibited markedly elevated rates of hepatic injury and shock, alongside reduced durations of antibiotic treatment (*P* < 0.001) and shorter hospital stays in comparison to survivors (Table 2).

Definitive etiological results were derived for 103 patients from medical records, owing to the constraints inherent in retrospective investigations. Pulmonary infections were the predominant site of infection, succeeded by bloodstream and urinary tract infections. Microbiologically, Acinetobacter baumannii was the primary pathogen, succeeded by Klebsiella pneumoniae and Pseudomonas aeruginosa (Supplementary Table S1).

Subsequent study of PCI patients revealed that the mortality group exhibited markedly elevated rates of left anterior descending artery lesions (*P* = 0.017) and intra-aortic balloon pump utilization during the procedure (*P* = 0.016) (Supplementary Table S2).

### 3.3 Identification of mortality risk factors in patients with ACS concomitant sepsis

**Figure 2:**
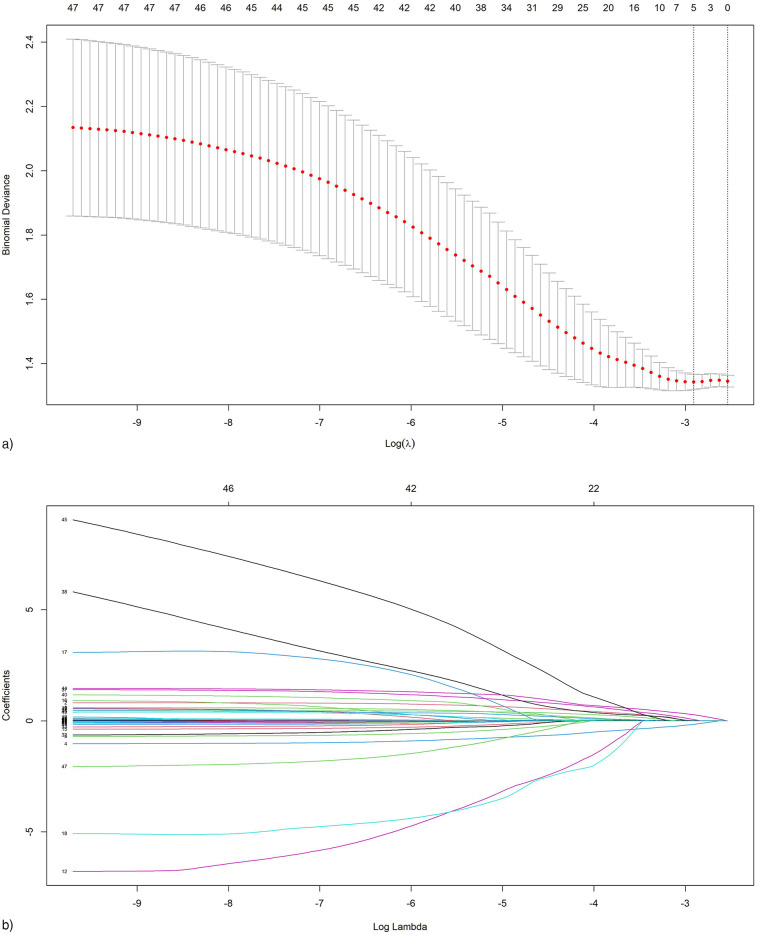
Feature selection using the least absolute shrinkage and selection operator (LASSO) regression. **(a)** Selection of tuning parameter (*λ*) in the LASSO regression using 10-fold cross-validation via minimum criteria. The partial likelihood binomial deviance is plotted vs. log (*λ*). At the optimal values log (*λ*), where features are selected, dotted vertical lines are set using the minimum criteria and the one standard error of the minimum criteria; **(b)** LASSO coefficient profiles for clinical features, each coefficient profile plot is produced vs. log (*λ*) sequence. Dotted vertical line is set at the nonzero coefficients selected via 10-fold cross-validation, where five nonzero coefficients are included.

A correlation analysis of the specified clinical indicators was conducted, resulting in the creation of a correlation heatmap that demonstrated multicollinearity among the variables (Supplementary Figure S1). To alleviate collinearity issues and discern principal predictors, we subsequently utilized least absolute shrinkage and selection operator (LASSO) regression for variable selection. Utilizing 10-fold cross-validation to ascertain the optimal penalty coefficient (λ = 0.055, aligned with the minimum mean squared error criterion), five variables with non-zero coefficients were ultimately identified (Figure 2): admission diagnosis, initial heart rate, initial neutrophil count, history of chronic gastritis, and history of CAD. The LASSO-selected variables were subsequently integrated into multivariate logistic regression for further validation, which ultimately identified STEMI diagnosis at admission, initial neutrophil count, and a history of CAD as independent risk factors for in-hospital mortality in patients with ACS concomitant sepsis (Table 3).

**Table 3:** Multivariate logistic regression analysis based on LASSO result.

Variables	Multivariate logistic regression result based on the LASSO regression result		Multivariate logistic regression result	
DIG	OR (95% CI)	*P* value	OR (95% CI)	*P* value
STEMI	1		2.081 (1.120–3.866)	**0**.0206*
UA	0.798 (0.281–2.264)	0.671	0.791 (0.422–1.484)	0.4661
NSTEMI	0.488 (0.251–0.95)	**0**.035*	1	
HR at admission	1.006 (0.995–1.018)	0.287		
NEUT at admission	1.035 (0.98–1.094)	**0**.**216**	1.05 (1–1.102)	**0**.0495*
Gastric disease	2.862 (0.591–13.874)	0.192		
CAD	2.806 (1.1–7.156)	**0**.031*	2.953 (1.173–7.436)	**0**.0215*

Asterisks denote statistical significance: *P < 0.05.

### 3.4 Development and verification of a nomogram

**Figure 3:**
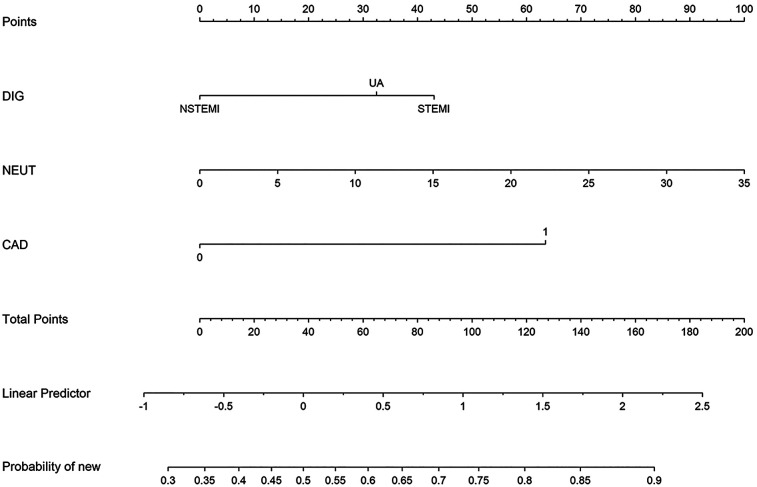
Nomogram for predicting mortality in patients with acute coronary syndrome complicated by sepsis. (To use the nomogram, locate the patient's value for each variable on the corresponding axis, draw a vertical line to the “Points” axis to determine the score for each variable, and sum all the scores. Locate the total points on the “Total Points” axis; a vertical line drawn downward to the “Probability” axis indicates the individual probability.).

A nomogram for predicting in-hospital mortality in patients with ACS concomitant sepsis was created based on the results of multivariate logistic regression (Figure 3). The receiver operating characteristic (ROC) curve analysis indicated the model's predictive efficacy, yielding an area under the curve (AUC) of 0.641 (95% CI: 0.564–0.718), a sensitivity of 0.656, and a specificity of 0.603 (Supplementary Figure S2). Calibration curves were produced, and the Hosmer-Lemeshow goodness-of-fit test demonstrated superior concordance between observed and predicted probabilities (*P* > 0.05), affirming robust model fit (Supplementary Figure S3).

## 4 Discussion

This study is the inaugural multicenter analysis aimed at assessing mortality risk factors and creating a nomogram prediction model for patients with ACS concomitant sepsis. An analysis of clinical data from 200 patients indicated a 61% in-hospital mortality rate among patients. Significant risk factors included STEMI diagnosis at admission, elevated initial neutrophil count, and a history of CAD. The developed nomogram model utilizing these three indicators exhibited an AUC of 0.641 and demonstrated satisfactory calibration (Hosmer-Lemeshow test *P* > 0.05). This discussion will offer a comprehensive analysis of the study's highlights, limitations, and clinical implications.

### 4.1 Pathophysiological mechanisms contributing to vulnerability in STEMI subtypes

Our findings indicate that among ACS subtypes, STEMI patients who developed sepsis had the highest mortality rate (66.4%) and were identified as an independent risk factor for mortality, presenting a more than 2-fold increased mortality risk compared to NSTEMI/UA patients. These observations closely correspond with established disease mechanisms.

STEMI, the most significant ACS subtype and a primary contributor to cardiovascular mortality, generally manifests as complete coronary occlusion, resulting in transmural myocardial ischemia and infarction (26, 27). Prior research indicates an in-hospital mortality rate of 5%–10% for STEMI patients (28, 29), who typically exhibit elevated peak troponin I levels, diminished left ventricular ejection fractions, and increased rates of ICU admission compared to NSTEMI/UA patients—factors that predispose them to the development of cytokine storms (30, 31). Infection is a significant independent predictor of long-term mortality in elderly patients with STEMI (32). STEMI patients demonstrate markedly higher levels of inflammatory markers, such as soluble suppression of tumorigenicity 2 (sST2) and interleukin-6 (IL-6), in comparison to NSTEMI patients. Increased sST2 may worsen myocardial injury and infection by obstructing the cardioprotective and immunomodulatory functions of the IL-33/ST2l pathway, thereby creating a detrimental “inflammation-infection” cycle (33–35).

The elevated revascularization rates in STEMI patients (75% of the deceased cohort underwent PCI in our study) may also heighten the risk of systemic infection due to invasive procedures such as catheterization. This supports Kohsaka et al.'s findings of a 12.9% incidence of catheter-related bloodstream infections among AMI patients experiencing cardiogenic shock (36).

Significantly, although admission diagnosis subtypes exhibited no notable differences in univariate analysis—possibly due to confounding variables or interactions among subtypes—current evidence indicates varying prognostic implications: STEMI primarily impacts short-term outcomes, while NSTEMI affects long-term prognosis (37, 38). Interestingly, an earlier study noted that among patients with ACS concomitant sepsis, the proportion of NSTEMI cases was significantly higher than that of STEMI (2). However, that study did not account for potential influences from the differing baseline incidence rates between NSTEMI and STEMI itself. To date, no subsequent research has provided further clarification on this specific direction. Future investigations concerning patients with ACS concomitant sepsis will require more rigorous prospective designs to address this gap. Furthermore, research utilizing multivariate machine learning techniques may more effectively tackle disease heterogeneity by leveraging multidimensional decision-making benefits.

### 4.2 Dual early-warning significance of neutrophil count

Although the initial multivariate regression did not reveal a significant correlation between neutrophil count and outcomes, it is important to note that, in pursuit of predictive modeling accuracy, neutrophil count may have influenced the coefficients of other variables in the initial regression while proving to be of substantial value in the final model. The results demonstrate that, relative to admission diagnosis and medical history, the initial neutrophil count at admission possesses greater predictive value for in-hospital mortality risk in patients with ACS concomitant sepsis, with each increase of 1*10^−9^/L correlating to a 5% rise in mortality risk. This predictive value is evident in two principal aspects.

Initially, the death group demonstrated markedly increased neutrophil counts upon admission, whereas intergroup disparities diminished following the sepsis diagnosis. Current research indicates that inflammatory responses contribute to atherosclerosis development, and neutrophil counts are significantly associated with disease severity and long-term outcomes in patients with myocardial infarction (39). This indicates that certain ACS patients may possess undetected low-grade infections, with increased admission neutrophils potentially signifying early indicators of subclinical infection or heightened inflammatory responses. Prior studies have identified increased neutrophil count as an independent risk factor for in-hospital mortality among patients experiencing cardiovascular events (40, 41). Elevated admission white blood cell counts independently associate with an increased risk of late cardiogenic shock in STEMI patients and higher 30-day mortality (42). Furthermore, white blood cell count serves as a pivotal variable for predicting the risk of infection in STEMI patients following PCI (43). Neutrophils, as fundamental effector cells of innate immunity (44), may experience overactivation, leading to heightened susceptibility to subsequent infections and exacerbation of organ injury associated with sepsis. Consequently, as a straightforward and accessible inflammatory marker, tracking early alterations in neutrophil counts may be especially beneficial for forecasting infection risk or prognosis in patients with ACS concomitant sepsis.

Secondly, neutrophils may engage in common pathways linking coronary events and infections. Neutrophil extracellular traps (NETs) have been shown to simultaneously contribute to plaque instability and microthrombosis associated with sepsis (45, 46). NETs facilitate the advancement of intracoronary thrombus via the activation of the platelet TLR4 pathway, exhibiting mechanistic parallels with sepsis-induced disseminated intravascular coagulation (47–49).

### 4.3 Coronary artery disease history as a “Second Hit” enhancer

Coronary artery disease is an independent risk factor for adverse outcomes, resulting in markedly diminished progression-free survival among survivors of cardiovascular events (50). This study identified a greater prevalence of CAD history in the death group, indicating an independent risk factor for mortality (2.9-fold increased risk compared to non-CAD patients). These findings indicate that CAD may not only contribute to the etiology of ACS but also aggravate organ injury associated with sepsis through various mechanisms, thereby deteriorating prognosis.

The classical theory asserts that coronary artery disease is a chronic inflammatory condition, characterized by significant inflammatory cell migration in atherosclerotic lipid cores and areas of plaque rupture (51). Chronic low-grade inflammation may induce dysfunction in the innate immune system (e.g., neutrophils), resulting in a “inflammatory priming” effect that can lead to either excessive activation or insufficient response during sepsis, thereby exacerbating organ damage and septic responses (52). Research indicates that in CAD patients with simultaneous infection, the severity of the infection is associated with the severity of coronary lesions, exhibits a prolonged duration compared to non-CAD patients, and may expedite the progression of atherosclerotic plaques (53–55).

Moreover, CAD patients often demonstrate dysfunction of coronary microcirculation, myocardial fibrosis, or left ventricular impairment. Systemic inflammation and hypotension induced by sepsis can lead to acute cardiac deterioration, which diminishes myocardial perfusion and exacerbates microcirculatory abnormalities, thereby worsening tissue hypoxia and organ dysfunction (56–58).

### 4.4 Infection and causative agents

The analysis of positive culture results in this study indicated that lower respiratory tract infections represented the largest proportion, a finding that aligns with sepsis-related clinical research data from the past decade, especially notable in community-acquired sepsis (23, 59). In ICUs, bloodstream infections exhibited elevated incidence rates, whereas urinary tract infections were significantly linked to indwelling catheters and geriatric patient demographics. Numerous studies on severe infections subsequent to cardiovascular events have identified pulmonary infections, urinary tract infections, and invasive catheter-related infections as the predominant types of infection (60).

In terms of pathogen distribution, Acinetobacter baumannii exhibited the highest detection rate, succeeded by Klebsiella pneumoniae and Pseudomonas aeruginosa, corroborating prior etiological profiles of severe infections following cardiovascular events. Historical research data indicate that Staphylococcus aureus is the predominant pathogen in AMI patients experiencing cardiogenic shock, succeeded by Klebsiella pneumoniae and Pseudomonas aeruginosa (61). A separate dataset indicates that Klebsiella pneumoniae, Staphylococcus aureus, and Pseudomonas aeruginosa are the most commonly identified species in bacterial cultures of STEMI patients following PCI (35). Current epidemiological studies on sepsis indicate that Gram-negative bacteria are the primary pathogens, with Acinetobacter baumannii prevailing in ICUs, while fungal infections show a consistent increase in ICUs.

The pathogen findings in this study were partially affected by bacterial resistance patterns and challenges in differentiating colonization from infection. While our observed distribution of pathogens and characteristics of infection sites align with previous studies on sepsis epidemiology and infections related to cardiovascular events, this resemblance indicates that the types and locations of infections may not serve as definitive mortality determinants in patients with ACS concomitant sepsis. Nonetheless, significant limitations must be recognized: this study cannot determine definitive correlations between infection sites, pathogen types, and drug resistance or pathogenicity; existing data cannot rule out potential confounding influences of colonizing bacteria; and the clinical relevance of pathogen findings necessitates further assessment that includes individual patient characteristics (e.g., immune status, comorbidities).

### 4.5 Model interpretation and optimization strategies

The evaluation of model performance was conducted through ROC curves and Hosmer-Lemeshow goodness-of-fit tests. The Hosmer-Lemeshow test demonstrated adequate calibration performance, supporting model generalizability across centers; however, the discriminative ability (AUC = 0.641) was suboptimal, warranting additional analysis in light of study design constraints.

Sample size limitations constitute the principal factor leading to inadequate AUC values. Predictive models generally necessitate compliance with the “10 events per variable” (EPV) guideline. This study, with 200 enrolled patients and 122 mortality events, could only accommodate 3–4 variables, thereby constraining the expansion of predictors. The limited sample size led to data sparsity in specific subgroups, undermining stability evaluations. Secondly, the omission of critical variables further reduced AUC performance. Dynamic alterations in myocardial injury markers may indicate the progression of injury and assist in prognostic assessment (61, 62); however, the standardization of measurement units and timepoints poses significant challenges. Despite the absence of significant intergroup differences in norepinephrine dosage in this study, hemodynamic parameters, such as point-of-care ultrasound data, possess recognized prognostic value in SICM (63, 64). Prior research links multidrug-resistant and extensively drug-resistant Acinetobacter baumannii, as well as polymicrobial infections, to increased mortality rates (65). Although our data identified Acinetobacter baumannii as the most prevalent pathogen, resistance patterns and virulence analysis results were not accessible for comprehensive assessment.

To mitigate these limitations, we propose the following optimization strategies: Firstly, increasing the sample size is essential, potentially through multinational collaborative cohorts sourced from public databases. Future prospective studies ought to standardize the timing of data collection. Secondly, model refinement should integrate dynamic indicators and innovative biomarkers (e.g., neutrophil fluctuation rates and vascular endothelial damage markers). Current research indicates that multiple neutrophil-related factors possess predictive significance for the risk of mortality in sepsis (66, 67). Neutrophil CD64, when integrated with CRP and SOFA scores, attains an accuracy of 0.93 in forecasting sepsis mortality (68, 69). Vascular endothelial injury indicators, as markers of coronary plaque instability, may aggravate the pathological processes of ACS through sepsis-related inflammatory cascades (70, 71). Ultimately, machine learning techniques such as XGBoost algorithms may aid in stratification and managing nonlinear relationships to further improve AUC values.

### 4.6 Clinical importance and practical relevance of the model

This predictive model exhibits substantial clinical utility as a swift bedside triage instrument, necessitating merely three readily accessible parameters within 24 h of admission and capable of executing risk assessment in under 10 min. Its utility in emergency triage can improve resource allocation efficiency. For example, high-risk patients with pre-existing CAD, a STEMI diagnosis at emergency admission, and neutrophil counts exceeding 10,000 cells per microliter should be prioritized for ICU admission over general wards.

Secondly, the model offers decision-making assistance for tailored interventions. Risk stratification utilizing predictive factor mechanisms facilitates graduated treatment intensification and targeted intervention strategies.

This study provides a basis for subsequent research. Neutrophil serve as an independent risk factor and represent potential therapeutic targets for mechanistic validation. Moreover, integrated predictive-therapeutic studies may inform future research trajectories, wherein randomized controlled trials of various pharmacological therapies could substantiate the model's efficacy in directing precision treatment.

## 5 Conclusion

This study constitutes the inaugural development of a nomogram prediction model for in-hospital mortality in patients with ACS concomitant sepsis. Three independent risk factors were identified from multicenter retrospective data (*n* = 200): STEMI diagnosis at admission (OR = 2.081), elevated admission neutrophil count (OR = 1.05), and a history of CAD (OR = 2.953). The model exhibited an AUC of 0.641 (95% CI: 0.564–0.718) with satisfactory calibration (Hosmer-Lemeshow test *P* > 0.05). The results emphasize the model's capacity for early risk assessment and clinical decision-making in this high-mortality patient demographic, while also indicating the necessity for additional validation and enhancement in larger prospective cohorts.

## Data Availability

The raw data supporting the conclusions of this article will be made available by the authors, without undue reservation.
