# Supplementary material for: Characteristics and risk factors for mortality in patients with acute coronary syndrome concomitant sepsis: a retrospective multicenter cohort study
Source: Front Cardiovasc Med. 2025 Nov 18;12:1703505. doi: 10.3389/fcvm.2025.1703505 (PMC12669186; doi:10.3389/fcvm.2025.1703505)
Supplement: Supplementary file 1 [file Table1.doc]

**Table S1** Comparison of etiological characteristics between the survival and death groups

| **Variables** | **Total**  **(*n* =103)** | **Died**  **(*n* =33)** | **Survived**  **(*n* =70)** | ***P* Value** |
| --- | --- | --- | --- | --- |
| Sites (%) |  |  |  |  |
| Sputum | 76 (73.8) | 25 (75.8) | 51 (72.9) | 0.942 |
| Catheter | 11 (10.7) | 2 (6.1) | 9 (12.9) | 0.484 |
| Urine | 26 (25.2) | 7 (21.2) | 19 (27.1) | 0.687 |
| Lavage Fluid | 1 (1.0) | 0 (0.0) | 1 (1.4) | 1 |
| Blood | 34 (33.0) | 7 (21.2) | 27 (38.6) | 0.128 |
| Pleural Effusion | 3 (2.9) | 1 (3.0) | 2 (2.9) | 1 |
| Ascites | 1 (1.0) | 0 (0.0) | 1 (1.4) | 1 |
| Secretions | 2 (1.9) | 0 (0.0) | 2 (2.9) | 0.829 |
| Feces | 3 (2.9) | 0 (0.0) | 3 (4.3) | 0.563 |
| Number of Positive Sites(%) |  |  |  |  |
| 1 | 1 (1.0) | 0 (0.0) | 1 (1.4) | 0.44 |
| 2 | 70 (68.0) | 26 (78.8) | 44 (62.9) |  |
| 3 | 16 (15.5) | 5 (15.2) | 11 (15.7) |  |
| 4 | 10 (9.7) | 2 (6.1) | 8 (11.4) |  |
| ≥5 | 6 (5.8) | 0 (0.0) | 6 (8.6) |  |
| Species(%) |  |  |  |  |
| Pulmonary Klebsiella | 31 (30.1) | 7 (21.2) | 24 (34.3) | 0.263 |
| Staphylococcus aureus | 14 (13.6) | 4 (12.1) | 10 (14.3) | 1 |
| Acinetobacter baumannii | 41 (39.8) | 14 (42.4) | 27 (38.6) | 0.875 |
| Pseudomonas aeruginosa | 17 (16.5) | 5 (15.2) | 12 (17.1) | 1 |
| Escherichia coli | 12 (11.7) | 3 (9.1) | 9 (12.9) | 0.821 |
| Enterococci | 14 (13.6) | 5 (15.2) | 9 (12.9) | 0.993 |
| Other Bacteria | 34 (33.0) | 9 (27.3) | 25 (35.7) | 0.532 |
| Fungi | 36 (35.0) | 16 (48.5) | 20 (28.6) | 0.079 |
| Mixed Fungal and Bacterial Infection | 28 (27.2) | 13 (39.4) | 15 (21.4) | 0.094 |
