# Supplementary material for: Characteristics and risk factors for mortality in patients with acute coronary syndrome concomitant sepsis: a retrospective multicenter cohort study
Source: Front Cardiovasc Med. 2025 Nov 18;12:1703505. doi: 10.3389/fcvm.2025.1703505 (PMC12669186; doi:10.3389/fcvm.2025.1703505)
Supplement: Supplementary file 2 [file Table2.doc]

**Table S2** Analysis of surgical situation in patients undergoing PCI

| **Variables** | **Total**  **(*n* =141)** | **Died**  **(*n* =59)** | **Survived**  **(*n* =82)** | ***P* Value** |
| --- | --- | --- | --- | --- |
| Arterial Access Route (%) |  |  |  | 0.598 |
| RA | 86 (61) | 38 (64) | 48 (59) |  |
| CFA | 44 (31) | 18 (31) | 26 (32) |  |
| Both | 11 (8) | 3 (5) | 8 (10) |  |
| LM (%) | 25 (18) | 7 (12) | 18 (22) | 0.186 |
| LAD (%) | 100 (71) | 35 (59) | 65 (79) | **0.017** |
| LCX (%) | 81 (57) | 30 (51) | 51 (62) | 0.241 |
| RCA (%) | 87 (62) | 38 (64) | 49 (60) | 0.7 |
| Stent length (mm) | 23 (0, 38) | 23 (14.5, 37) | 23.5 (0, 39.5) | 0.694 |
| IABP in PCI (%) | 32 (23) | 7 (12) | 25 (30) | **0.016** |
| DAPT (%) |  |  |  | 0.960 |
| ASP+CLP | 116 (82.3) | 49 (83.1) | 67 (81.7) |  |
| ASP+TCA | 25 (17.7) | 10 (16.9) | 15 (18.3) |  |

Abbreviations: RA, Radial Artery, CFA, Common Femoral Artery, LM, Left Main Coronary Artery, LAD, Left Anterior Descending Artery, LCX, Left Circumflex Artery, RCA, Right Coronary Artery, IABP,Intra-Aortic Balloon Pump, DAPT, Dual Antiplatelet Therapy, ASP,Aspirin plus, CLP,Clopidogrel, TCA,Ticagrelo
