# Supplementary figures and images for: Characteristics and risk factors for mortality in patients with acute coronary syndrome concomitant sepsis: a retrospective multicenter cohort study
Source: Front Cardiovasc Med. 2025 Nov 18;12:1703505. doi: 10.3389/fcvm.2025.1703505 (PMC12669186; doi:10.3389/fcvm.2025.1703505)

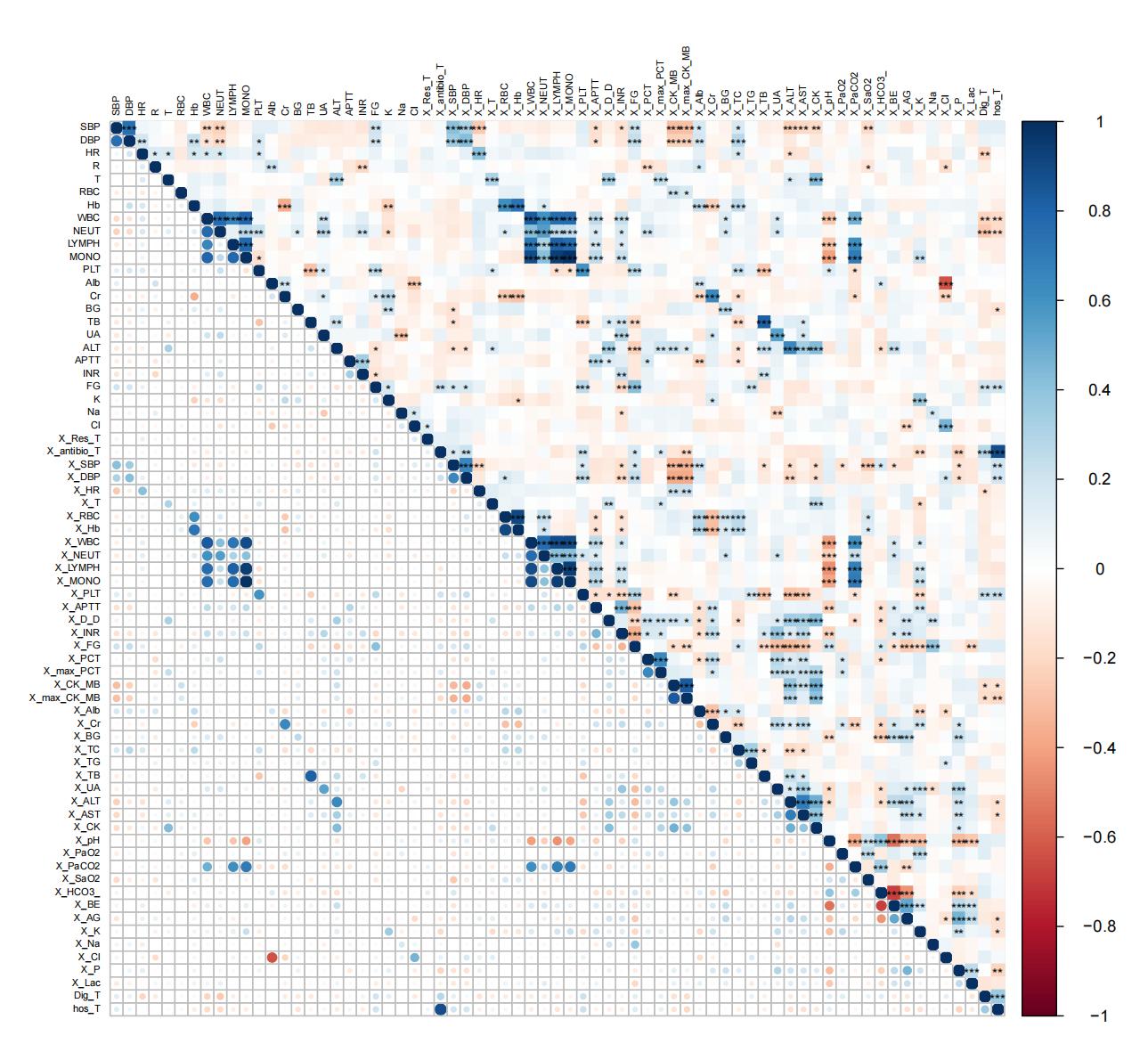

Supplement: Supplementary file 3 [file Image1.jpeg]

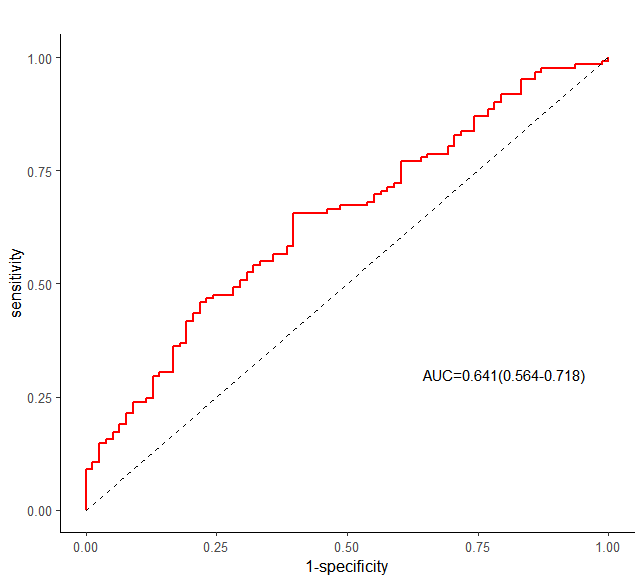

Supplement: Supplementary file 4 [file Image2.jpeg]

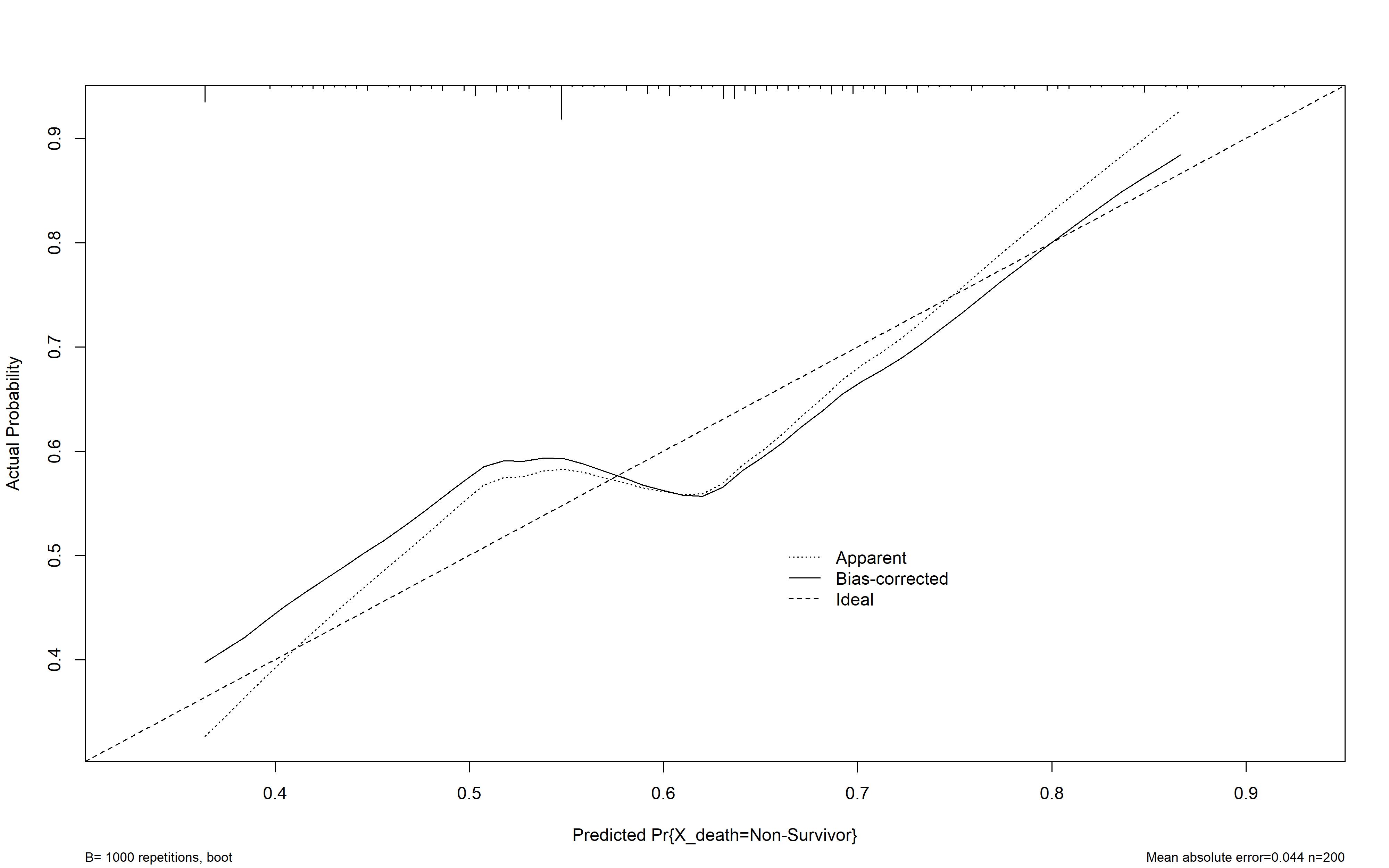

Supplement: Supplementary file 5 [file Image3.jpeg]
